# Human β-defensin 2 plays a regulatory role in innate antiviral immunity and is capable of potentiating the induction of antigen-specific immunity

**DOI:** 10.1186/s12985-018-1035-2

**Published:** 2018-08-08

**Authors:** Ju Kim, Ye Lin Yang, Sun-Hee Jang, Yong-Suk Jang

**Affiliations:** 1 Department of Molecular Biology and the Institute for Molecular Biology and Genetics, Chonbuk National University, Jeonju, 54896 Korea; 2 Department of Bioactive Material Sciences and Institute of Bioactive Materials, Chonbuk National University, Jeonju, 54896 Korea

**Keywords:** Adjuvant, Antigen, Antibody, Human β-defensin, MERS-CoV

## Abstract

**Background:**

Antimicrobial peptides (AMPs) are primarily known for their innate immune defense against invading microorganisms, including viruses. In addition, recent research has suggested their modulatory activity in immune induction. Given that most subunit vaccines require an adjuvant to achieve effective immune induction through the activation of innate immunity, AMPs are plausible candidate molecules for stimulating not only innate immune but also adaptive immune responses.

**Results:**

In this study, we investigated the ability of human β-defensin (HBD) 2 to promote antiviral immunity in vitro and in vivo using a receptor-binding domain (RBD) of Middle East respiratory syndrome-coronavirus (MERS-CoV) spike protein (S RBD) as a model antigen (Ag). When HBD 2-conjugated S RBD was used to treat THP-1 human monocytic cells, the expression levels of antiviral (IFN-β, IFN-γ, MxA, PKR, and RNaseL) and primary immune-inducing (NOD2, TNF-α, IL-1β, and IL-6) molecules were enhanced compared to those expressed after treatment with S RBD only. The expression of chemokines capable of recruiting leukocytes, including monocytes/macrophages, natural killer cells, granulocytes, T cells, and dendritic cells, was also increased following HBD 2-conjugated S RBD treatment. More important, immunization of mice with HBD 2-conjugated S RBD enhanced the immunogenicity of the S RBD and elicited a higher S RBD-specific neutralizing antibody response than S RBD alone.

**Conclusions:**

We conclude that HBD 2 activates the primary antiviral innate immune response and may also mediate the induction of an effective adaptive immune response against a conjugated Ag.

## Background

In general, vaccination materials consist of a specific antigen (Ag) and an adjuvant capable of potentiating the immunogenicity of the Ag to achieve efficient Ag-specific adaptive immunity [1]. Vaccines made up of live attenuated and/or killed whole pathogens usually contain endogenous adjuvants, such as bacterial cell wall components, genomic nucleic acids, and various pathogen-derived materials, that act as pathogen-associated molecular patterns and are sufficient to induce Ag-specific adaptive immunity by potentiating immunogenicity through the activation of innate immunity [2, 3]. However, subunit vaccines that utilize recombinant and/or purified Ags usually lack these endogenous innate immune stimulators. Consequently, the addition of exogenous materials with adjuvant activity is required to mimic natural infection to draw effective pathogenic Ag-specific adaptive immunity [4].

The innate immune response includes the production of interferons (IFNs), complements, and antimicrobial peptides (AMPs) and is crucial for controlling infectious diseases and inducing adaptive immunity [5]. AMPs have been proposed as multifunctional peptides that participate in the elimination of pathogenic microorganisms, including bacteria, fungi, and viruses [6]. In particular, defensins, one of the major AMP families in mammals, contribute to the antimicrobial innate immune response by disrupting the cell membranes of pathogens [7]. Six α-defensins and 31 β-defensins are expressed in humans. Human β-defensins (HBDs), unlike α-defensins, are produced in a wide variety of epithelial tissues, including skin and mucosa, and cells, including phagocytic cells and mucosal epithelial cells [7]. The expression of defense genes is tightly regulated by cytokines as part of the host defense and is suppressed by various virulent factors of pathogens [6]. It is important to note that AMPs were recently reported to modulate adaptive immunity by triggering the recruitment and activation of immune cells via various pathways linked with innate immunity [8]. For example, HBDs are chemotactic for immature dendritic cells (DCs) and memory T cells to the site of pathogen invasion by interacting with CCR6 and promote the adaptive immune response by recruiting immune cells [9, 10]. However, their antiviral and immune-modulatory functions against viral infection have not been clearly elucidated in crucial innate immune cells, such as neutrophils and macrophages.

Here we investigated the use of AMPs as an adjuvant to stimulate the induction of not only antiviral innate immunity but also Ag-specific adaptive immune responses using HBD 2 and the receptor-binding domain (RBD) of Middle East respiratory syndrome-coronavirus (MERS-CoV) spike (S) protein (S RBD) as a model Ag. We assessed the immune-modulatory activity of HBD 2 in macrophage-like THP-1 cells to determine the active participation of HBD 2 in innate immunity against virus infection. In addition, we confirmed the adjuvant activity of HBD 2 in vivo by determining the level of Ag-specific immune response induction after the administration of HBD 2-conjugated S RBD.

## Methods

### Experimental animals and materials

Six- to eight-week-old female C57BL/6 mice were purchased from the Koatech Laboratory Animal Center (Pyeongtaek, Korea) and housed under specific pathogen-free conditions with water and food provided ad libitum. Animal experiments were approved by the Institutional Animal Care and Use Committee of Chonbuk National University (Approval No. CBNU 2017–0055) and followed the guidelines suggested by the committee.

THP-1 (ATCC® TIB-202™) and Vero E6 (ATCC® CRL-1586™) cells were obtained from the American Type Culture Collection (Manassas, VA, USA). Huh-7 cells (KCLB No. 60104) were obtained from the Korean Cell Line Bank (Seoul, Korea). MERS-CoV (1–001-MER-IS-2015001) was obtained from the Korean Center for Disease Control and Prevention (KCDC). All experiments using MERS-CoV were performed in accordance with the World Health Organization’s recommendations under biosafety level 3 conditions in a biosafety level 3 facility in the Korea Zoonosis Research Institute at Chonbuk National University. Unless otherwise specified, the chemicals and laboratory wares used in this study were obtained from Sigma Chemical Co. (St. Louis, MO, USA) and SPL Life Sciences (Pocheon, Korea), respectively.

### Recombinant protein production and cell culture

Gene recombination, expression, and the purification of recombinant MERS-CoV S RBD with or without HBD 2 at the C terminus of the RBD (residues 291–725) of the S1 domain were performed as described previously with minor modifications [11]. Briefly, the gene encoding S RBD was synthesized with codon optimization based on the MERS-CoV S protein sequence (GenBank: AKL59401.1; GenScript, Piscataway, NJ, USA). The S RBD gene with the HBD 2 gene at its 3′ terminus was amplified by polymerase chain reaction (PCR) using the forward primer (5′-GAG CTC AAG TAT TAT TCT ATC ATT CCT-3′, where the underlined letters represent the SacI restriction site) with the HBD 2 gene together with the reverse primer (5′-TCT AGA *TCA TGG CTT TTT GCA GCA TTT TGT TCC AGG GAG ACC ACA GGT GCC AAT TTG TTT ATA CCT TCT AGG GCA AAA GAC TGG ATG ACA TAT GGC TCC ACT CTT AAG GCA GGT AAC AGG ATC GCC TAT ACC* CTC TAC GAA CAA AGA GGA-3′, where the underlined and italicized letters represent the XbaI restriction site and the HBD 2 sequence, respectively). Amplified genes were cloned into the pColdII *Escherichia coli* expression vector (TaKaRa Bio, Shiga, Japan). Recombinant proteins were purified by Ni-NTA Superflow (Qiagen, Valencia, CA, USA) for proteins with an N-terminal His tag according to the manufacturer’s instructions. Any residual endotoxin contamination was filtered out using a Sartobind Q75 membrane chromatography system (Sartorius, Goettingen, Germany), such that the final endotoxin content of recombinant proteins was below 0.5 EU per μg proteins, determined using an LAL chromogenic endotoxin quantification kit (Thermo-Fischer Scientific, Rockford, IL, USA).

THP-1 cells were cultured in RPMI medium (Welgene, Gyeongsan, Korea) supplemented with 10% fetal bovine serum (FBS; Gibco, Grand Island, NY, USA) at 37 °C in a humidified CO_2_ incubator. THP-1 cells were treated with phorbol-12-myristate-13-acetate (1 μg/mL for 1 × 10^6^ cells) for 2–3 days to differentiate into monocyte-derived macrophage cells [12]. The cells were replenished with fresh media and maintained for 3 days and then treated with recombinant protein (1 μg/mL per 1 × 10^6^ cells). The cells were harvested 6 h and 24 h after recombinant protein treatment and subjected to quantitative real-time PCR (qRT-PCR) to assess the expression levels of the target genes. At the same time, cell culture supernatants were collected and subjected to expression profiling for cytokine and chemokine proteins, which are related to innate immunity, using a LEGENDplex human pro-inflammatory chemokine and Type I/II/III interferon panel (BioLegend, San Diego, CA, USA), according to the manufacturer’s protocol.

### RNA extraction and qRT-PCR

We performed RNA extraction using TRIzol® reagent (Thermo-Fisher Scientific, Waltham, MA, USA) according to the manufacturer’s instructions. We converted prepared RNA into cDNA using an MMLV Reverse Transcription Kit (Promega, Fitchburg, WI, USA). We quantified gene expression via qRT-PCR using the QuantiTect SYBR Green PCR Kit (Qiagen, Hilden, Germany) with an ABI 7500 system (Applied Biosystems, Foster City, CA, USA) using 50 ng first-strand cDNA under the following conditions: 95 °C for 5 min followed by 40 amplification cycles at 95 °C for 15 s, 55 °C for 30 s, and 72 °C for 30 s. We normalized the expression level of each gene to that of β-actin (hACTB) via a relative quantification method using 7500 FAST software version 2.0.6 (Applied Biosystems). The gene-specific primer sets used to amplify each gene are listed in Table 1.

**Table 1:** Primer sequences used for qRT-PCR to measure the transcript levels of specific genes

Gene	Primer sequences
hACTB	F: 5′-CCA ACC GCG AGA AGA TGA-3′
	R: 5′-TCC ATC ACG ATG CCA GTG-3′
CXCL-1	F: 5′-ATT CAC CCC AAG AAC ATC CA-3′
	R: 5′-TGG ATT TGT CAC TGT TCA GCA-3′
CXCL-10	F: 5′-AGT GGA TGT TCT GAC CCT GCT TCA-3′
	R: 5′-TGG GCC CCT TGG GAG GAT GG-3′
IFN-β	F: 5′-TTT CAG TGT CAG AAG CTC CT-3′
	R: 5′-TGG CCT TCA GGT AAT GCA GA-3′
IFN-γ	F: 5′-CCA ACG CAA AGC AAT ACA TGA-3′
	R: 5′-CCT TTT TCG CTT CCC TGT TTT-3′
IL-1β	F: 5′-CCT GTC CTG CGT GTT GAA AGA-3′
	R: 5′-GGG AAC TGG GCA GAC TCA AA-3′
IL-6	F: 5′-TGG CTG AAA AAG ATG GAT GCT-3′
	R: 5′-TCT GCA CAG CTC TGG CTT GT-3′
MCP-1	F: 5′-ACT GAA GCT CGC ACT CTC-3′
	R: 5′-CTT GGG TTG TGG AGT GAG-3′
MIP-1α	F: 5′-CAG CAG ACA GTG GTC AGT CC-3′
	R: 5′-TTC TGA GCA GGT GAC GGA AT-3′
MxA	F: 5′-CTG TGG CCA TAC TGC CAG GA-3′
	R: 5′-ACT CCT GAC AGT GCC TCC AA-3′
NOD2	F: 5′-CGG CGT TCC TCA GGA AGT AC-3′
	R: 5′-ACC CCG GGC TCA TGA TG-3′
Protein kinase R	F: 5′-CAG GCA CGA CAA GCA TAG AA-3′
	R: 5′-CTA CTC CCT GCT TCT GAC GG-3′
RANTES	F: 5′-CCT CAT TGC TAC TGC CCT CT-3′
	R: 5′-GGT GTG GTG TCC GAG GAA TAT-3′
RNase L	F: 5′-GCA GAA ATG CCT TGA TCC AT-3′
	R: 5′-AGT CTT CAG CAG GAG GGT GA-3′
TNF-α	F: 5′-GGA GAA GGG TGA CCG ACT CA-3′
	R: 5′-CTG CCC AGA CTC GGC AA-3′
upE	F: 5′-GCC TCT ACA CGG GAC CCA TA-3′
	R: 5′-GCA ACG CGC GAT TCA GTT-3′
Vero E6 β-actin	F: 5′-ATC GTG CGT GAC ATT AAG GAG-3′
	R: 5′-AGG AAG GAA GGC TGG AAG AG-3′

F and R represent the forward and reverse primer sequences, respectively

### Immunization of mice and sample collection

C57BL/6 mice were immunized subcutaneously at the base of the tail and intramuscularly in the hind leg with 10 μg/mouse of each recombinant protein dissolved in 50 μL phosphate-buffered saline (PBS) emulsified with an equal volume of Freund’s complete adjuvant and boosted once with the same immunogen emulsified with Freund’s incomplete adjuvant 10 days after the first immunization. Control mice were immunized with the inoculum prepared identically but with PBS only. Sera were collected 3 days after boost immunization to assess the MERS-CoV S RBD-specific antibody (Ab) response.

### Enzyme-linked immunosorbent assay (ELISA)

The level of the MERS-CoV S RBD-specific Ab in mouse sera was determined by ELISA. Briefly, a 96-well ELISA plate (Thermo-Fisher Scientific) was precoated with S RBD protein (2 μg/mL) overnight at 4 °C and blocked with 5% nonfat dried milk at 37 °C for 2 h. After the addition of serially diluted sera to each well, the plate was incubated at 37 °C for 1 h, followed by four washes with PBS containing Tween 20. Bound Abs were incubated with alkaline phosphate-conjugated anti-mouse IgG at 37 °C for 1 h and the reaction was visualized with the addition of *p*-nitrophenyl phosphate substrate. We measured color development using reading the absorbance at 405 nm on an ELISA plate reader (SPECTROstar Nano, BMG Labtech, Ortenberg, Germany).

### Virus neutralization assay

Vero E6 cells were used to propagate MERS-CoV and were grown in Dulbecco’s Modified Eagle’s Medium (DMEM; Welgene) supplemented with 10% FBS (Gibco) at 37 °C in a humidified CO_2_ incubator. MERS-CoV was passed six times in Vero E6 cells and used to assess the neutralizing potential of each recombinant protein. Briefly, sera obtained from mice immunized with each recombinant protein were diluted 50-fold and incubated for 1 h at room temperature with 1 μg S RBD protein before being transferred to Huh-7 cells grown in confocal dishes. For the immunofluorescence assay, Huh-7 cell monolayers were fixed with 4% paraformaldehyde and treated with a premixture of sera and S RBD protein. Penta-His Ab coupled with Alexa Fluor® 488 (Qiagen) was used as a secondary Ab. Cells were covered with SlowFade Gold Antifade Reagent (Invitrogen, Grand Island, NY, USA), and slides were observed under a confocal laser scanning microscope (CLSM, LSM 510, Carl Zeiss, Thornwood, NY, USA). Additionally, a receptor binding inhibition assay was performed with MERS-CoV-susceptible Vero E6 cells as described above, and median fluorescence intensity was measured using a CytoFLEX flow cytometer (Beckman-Coulter, Indianapolis, IN, USA).

We assessed the neutralizing capacity of each individual serum sample by determining the viral loads in MERS-CoV-infected cells by quantifying the level of upstream E (upE) gene expression using qRT-PCR [13, 14]. Briefly, 100-fold diluted mouse immune sera were incubated for 1 h at room temperature with 10^3^ plaque-forming units (PFUs) of MERS-CoV and transferred to Vero E6 cells (1 × 10^6^ cells/well) grown in a 6-well tissue culture plate. After 24 h of incubation, we extracted total RNA and performed qRT-PCR as described previously using the primer set listed in Table 1 to measure the level of MERS-CoV upE gene expression.

### Statistical analyses

Statistical analyses were performed using Prism 7 (GraphPad, San Diego, CA, USA). Data are presented as means ± standard deviations (SDs). The statistical significance of numerical data was analyzed using two-way analysis of variance (ANOVA), and *p* < 0.05 was considered statistically significant.

## Results

### Stimulation of genes related to innate immunity in macrophage-like THP-1 cells by HBD 2-conjugated ag treatment

**Fig. 1:**
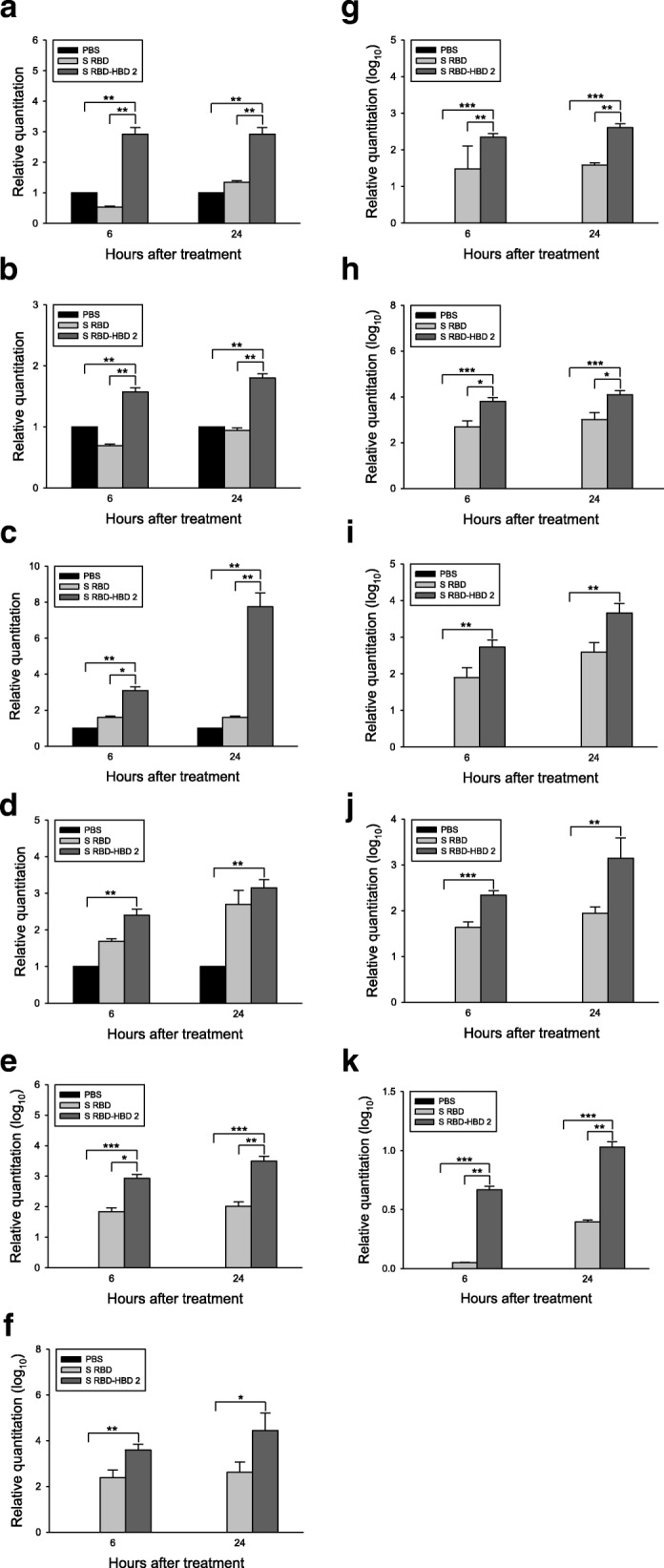
Quantitative analysis of cytokine and chemokine gene expression associated with innate antiviral activity in HBD 2-treated THP-1 cells. THP-1 cells were stimulated with 1 μg/mL recombinant S RBD protein with (S RBD-HBD 2) or without (S RBD) HBD 2 conjugation, and the transcript levels of (**a**) IFN-β, (**b**) IFN-γ, (**c**) MxA, (**d**) TNF-α, (**e**) IL-1β, (**f**) IL-6, (**g**) CXCL-1, (**h**) CXCL-10, (**i**) MCP-1, (**j**) MIP-1α, and (**k**) RANTES were measured 6 h and 24 h after treatment via quantitative real-time polymerase chain reaction (qRT-PCR) as described in Materials and Methods. qRT-PCR was performed twice, and the values were normalized to that of the internal control (hACTB). The relative quantification level using the value of phosphate-buffered saline (PBS)-treated control cells as a basal reference level for comparison is shown as the mean ± standard deviation (SD). *p < 0.05, ** *p* < 0.01, and *** *p* < 0.001 indicate significant differences between groups

Macrophages are one of the crucial immune cells involved in antiviral innate immunity. To assess the influence of HBD 2 on antiviral innate immune response induction, we determined the expression levels of mRNAs encoding cytokines/chemokines associated with antiviral innate immunity by qRT-PCR in macrophage-like THP-1 cells treated with recombinant S RBD with or without HBD 2 conjugation for 6 h and 24 h (Fig. 1). Specifically, we analyzed cytokine genes associated with classic antiviral immunity (IFN-β, IFN-γ, and MxA) and acquired immune induction (TNF-α, IL-1β, and IL-6) as well as chemokines (CXCL-1, CXCL-10, RANTES, MCP-1, and MIP-1α). These cytokines and chemokines were selected based on their known functions in vitro and in vivo using various viruses associated with respiratory diseases, including severe acute respiratory syndrome-coronavirus (SARS-CoV) and MERS-CoV [15–17].

HBD 2-conjugated RBD treatment in THP-1 cells significantly (*p* < 0.05 and *p* < 0.01) promoted the early induction (6 h and 24 h) of IFN-β (type I IFN), MxA (type I IFN-induced molecule with broad antiviral activity), and IFN-γ (type II IFN) transcripts compared to treatment with S RBD only without HBD 2 conjugation (Fig. 1a–c). The transcript level of MxA was substantially elevated 6 h after HBD 2-conjugated Ag treatment and remained strongly induced 24 h after Ag treatment (Fig. 1c). In addition, the levels of all cytokine and chemokine genes tested were significantly higher in HBD 2-conjugated Ag-treated THP-1 cells than in control PBS-treated cells (Fig. 1d–k). It is important to note that the levels of IL-1β, CXCL-1, CXCL-10, and RANTES were significantly higher in HBD 2-conjugated Ag-treated THP-1 cells than in THP-1 cells treated with S RBD alone (Fig. 1e, g, h, and k). In addition, the transcript levels of the analyzed genes increased as the treatment time increased from 6 h to 24 h. We speculate that HBD 2-conjugated S RBD treatment enhances the expression of well-known innate response-inducing cytokines and chemokines to promote the recruitment of leukocytes and is capable of controlling primary and adaptive immunity at the site of pathogen infection.

### Enhanced expression of interferon and chemokine proteins involved in innate immunity by HBD 2-conjugated Ag treatment

**Fig. 2:**
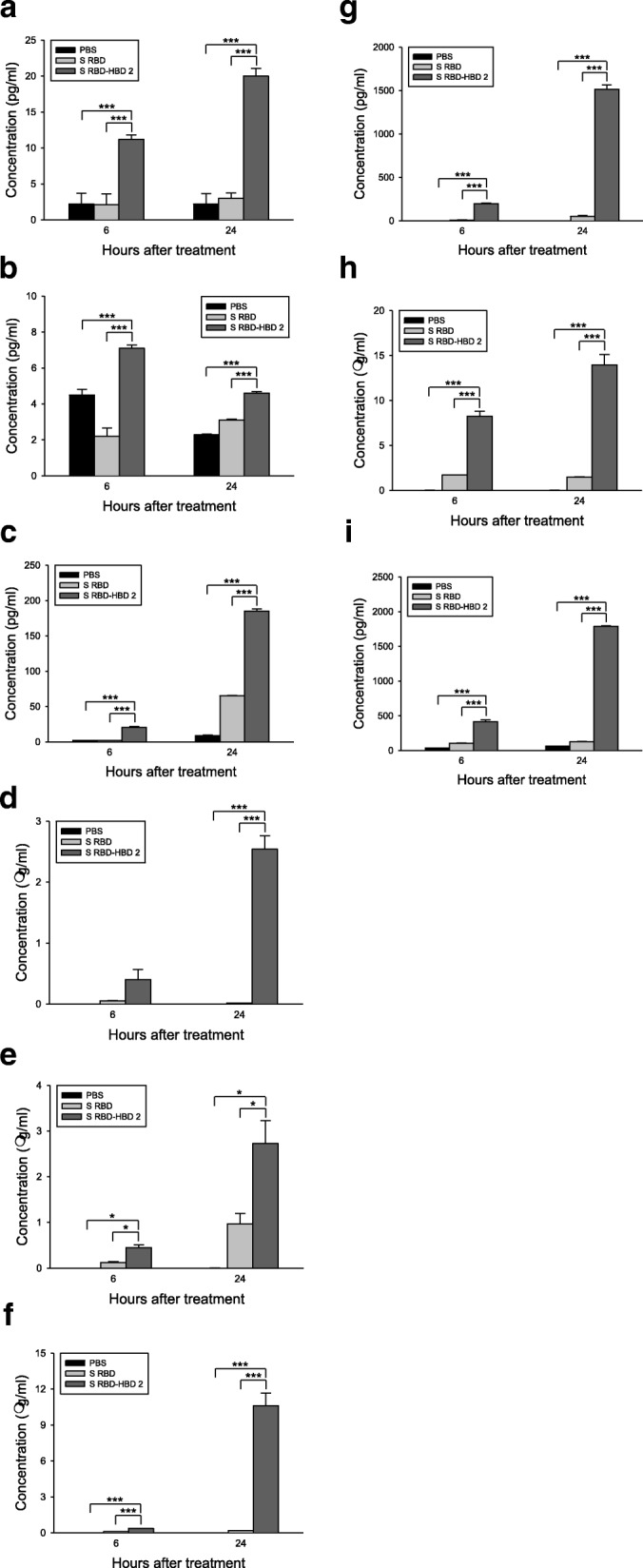
Quantitative analysis of type I/II/III interferon and chemokine profiles in HBD 2-conjugated S RBD-treated THP-1 cells. Cells were stimulated with 1 μg/mL recombinant S RBD protein with (S RBD-HBD 2) or without (S RBD) HBD 2 conjugation, and protein levels of (**a**) IFN-β, (**b**) IFN-γ, (**c**) IFN-λ, (**d**) CXCL-1, (**e**) CXCL-10, (**f**) MCP-1, (**g**) MIP-1α, (**h**) MIP-1β, and (**i**) RANTES were measured from culture supernatant by cytometric bead assay, as described in Materials and Methods, 6 h and 24 h after treatment. The reactions were performed in duplicate for every target protein in each experiment. The concentrations of each protein in the PBS, S RBD, and S RBD-HBD 2 supernatants were determined by comparison with the standard curve for target proteins. Means ± SDs are shown for each time point. * *p* < 0.05 and *** *p* < 0.001 indicate significant differences between groups

To confirm the enhanced expression of genes related to innate immunity by HBD 2-conjugated S RBD treatment, we determined the expression levels of cytokine and chemokine proteins involved in innate immunity (Fig. 2). We first examined the protein levels of type I/II/III interferons counteracting virus infection. As found in the qRT-PCR results, HBD 2-conjugated S RBD treatment of THP-1 cells significantly (*p* < 0.001) promoted IFN-β, IFN-γ, and IFN-λ expression compared with that of cells treated with S RBD without HBD 2-conjugation (Fig. 2a–c). The protein levels of primary chemokines leading to the initiation of innate immune response and changeover to adaptive immune response were also determined in THP-1 cells treated with S RBD, with or without HBD2 conjugation (Fig. 2d–i). Chemokine expression profiles were also consistent with the qRT-PCR results; significantly enhanced chemokine protein expression was observed in HBD 2-conjugated S RBD-treated THP-1 cells compared with that in cells treated with S RBD alone.

### Increased expression of genes involved in the antiviral response in HBD 2-conjugated Ag-stimulated THP-1 cells

**Fig. 3:**
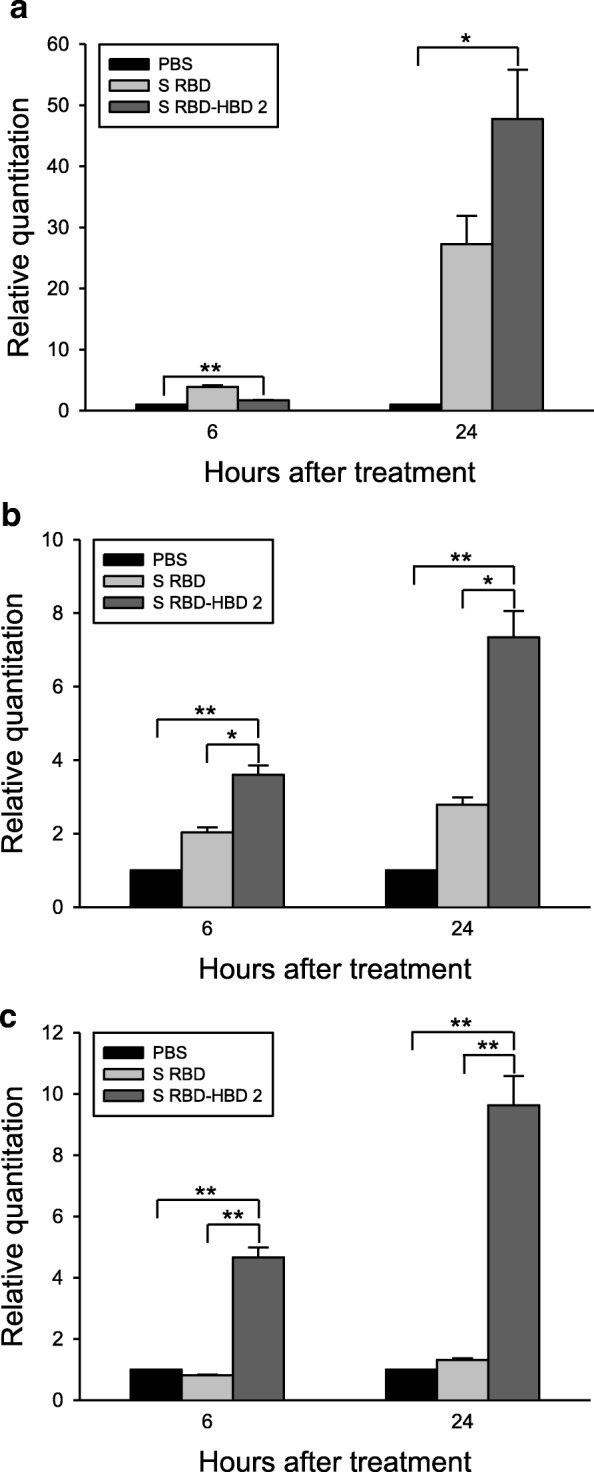
Quantitative analysis of antiviral gene expression in HBD 2-treated THP-1 cells. THP-1 cells were stimulated with 1 μg/mL recombinant S RBD protein with (S RBD-HBD 2) or without (S RBD) HBD 2 conjugation, and the transcript levels of (**a**) PKR, (**b**) RNaseL, and (**c**) NOD2 were measured 6 h and 24 h after treatment via qRT-PCR as described in Materials and Methods. qRT-PCR was performed twice, and values were normalized to that of the internal control (hACTB). The relative quantification level using the value of PBS-treated control cells as a basal reference level for comparison is shown as the mean ± SD. * *p* < 0.05 and ** *p* < 0.01 indicate significant differences between groups

In addition to MxA, an inhibitor of RNA virus replication, we analyzed the expression of genes involved in virus gene expression (Fig. 3). These genes included PKR, double-stranded RNA-dependent protein kinase; RNase L, an endogenous ribonuclease; and NOD2. PKR gene expression was significantly higher in HBD 2-conjugated Ag-treated THP-1 cells than in control PBS-treated cells; however, the difference between THP-1 cells treated with either HBD 2-conjugated or non-conjugated Ag-treated cells was not statistically significant, although HBD 2-conjugated Ag treatment upregulated the expression of PKR in THP-1 cells 24 h after treatment (Fig. 3a). It is interesting that the transcript levels of RNaseL and NOD2 were significantly higher in HBD 2-conjugated S RBD-treated cells 6 h after treatment than in cells treated with S RBD without HBD 2 conjugation, and this significant difference was maintained 24 h after treatment (Fig. 3b and c). More important, HBD 2-conjugated Ag treatment provoked a rapid and strong induction of NOD2 expression in THP-1 cells compared to treatment with S RBD alone (Fig. 3c). These results indicate that gene expression of antiviral innate and adaptive immune response-associated molecules is efficiently activated in THP-1 cells by responding to HBD 2.

### Enhanced induction of the Ag-specific immune response using HBD 2-conjugated ag

**Fig. 4:**
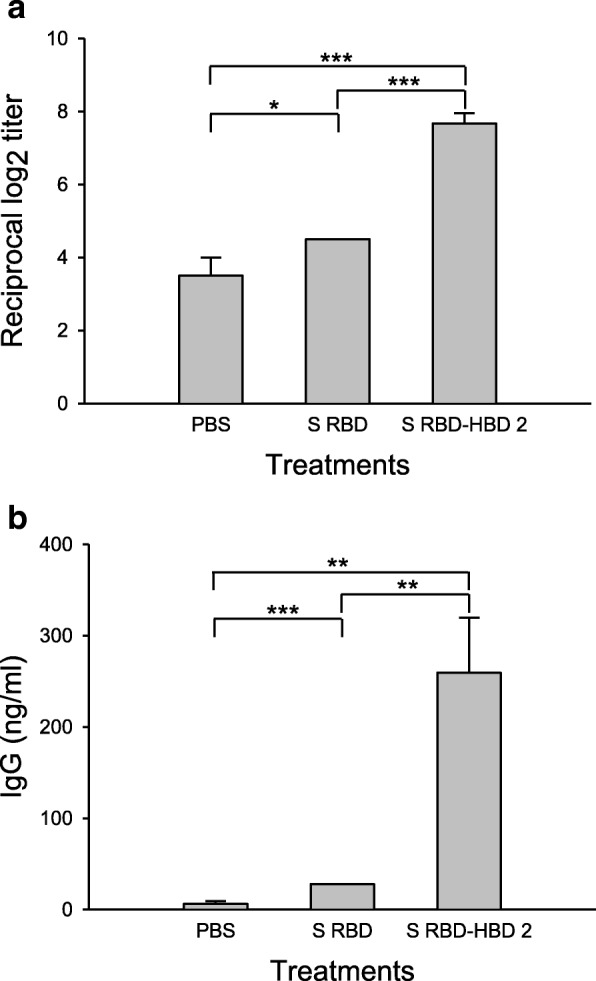
Quantitative analysis of the S RBD-specific antibody (Ab) response induced by the HBD 2-conjugated antigen (Ag). Mice were immunized with 10 μg/mouse of the indicated Ag and sera were collected 3 days after boost immunization. The level of the S RBD-specific Ab was measured via enzyme-linked immunosorbent assay, and data are represented as the mean ± SD (from triplicates): (**a**) S RBD-specific IgG endpoint titers and (**b**) amount of S RBD-specific IgG. * *p* < 0.05, ** *p* < 0.01, and *** *p* < 0.001 indicate significant differences between groups

Next we analyzed whether HBD 2 was capable of promoting the induction of the adaptive immune response specific to HBD 2-conjugated S RBD (Fig. 4). When we determined the level of the S RBD-specific Ab in sera induced 3 days after boost immunization, we found that the injection of HBD 2-conjugated S RBD induced a significantly (*p* < 0.001) higher titer (7.67 ± 0.29) of the S RBD-specific Ab compared to that (4.5 ± 0.01) induced after the injection of S RBD without HBD 2 conjugation (Fig. 4a). The S RBD-specific IgG level was significantly (*p* < 0.01) higher (259.2 ± 60.5 ng/mL) in sera obtained from mice injected with HBD 2-conjugated S RBD compared to that (27.7 ± 0.3 ng/mL) in mice injected with control S RBD without HBD 2 conjugation (Fig. 4b). These results suggest that HBD 2 conjugation is capable of mediating conjugated Ag-specific adaptive immune response induction and that HBD 2 exerts adjuvant activity on specific immune induction against the HBD 2-conjugated Ag.

### Ag-specific effector function of the immune response induced by the HBD 2-conjugated Ag

**Fig. 5:**
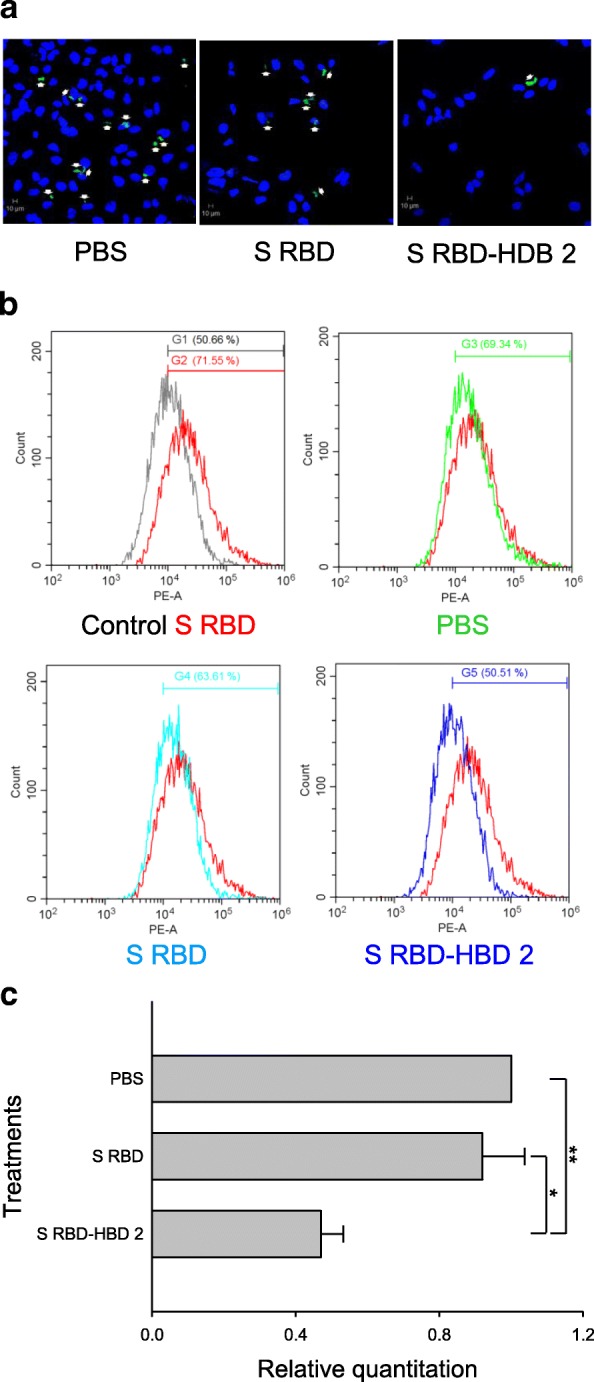
Neutralizing activity of the Ab induced by HBD 2-conjugated Ag injection against the binding of S RBD to MERS-CoV receptor-expressing cells. (**a**, **b**) Serum neutralizing activity and (**c**) in vitro inhibitory activity against MERS-CoV infection were determined by Ab-mediated receptor-binding inhibition assay. (**a**) Huh-7 cells were treated with S RBD that had been incubated with 50-fold diluted sera drawn from mice immunized with the indicated Ag. A specific monoclonal Ab against S RBD was used for indirect immunofluorescence, and slides were analyzed by confocal microscopy. Nuclei were stained with DAPI (blue), whereas the S RBD signal was stained with an Alexa Fluor 488-conjugated secondary Ab (green, arrows). Representative fields were observed at 200× magnitude, and the scale bar represents 10 μm. (**b**) Vero E6 cells were treated with S RBD alone (red line in upper left panel) or with S RBD that had been incubated with 50-fold-diluted sera drawn from mice immunized with either PBS (green line in upper right panel), S RBD alone (sky-blue line in lower left panel), or HBD 2-conjugated S RBD (blue line in lower right panel). Grey line in upper left panel represents the control Ab treatment result. (**c**) Viral upE gene transcript level in Vero E6 cells 24 h after treatment with MERS-CoV (10^3^ PFU) that had been incubated with 100-fold diluted sera drawn from mice immunized with the indicated Ag. qRT-PCR was performed as described in Materials and Methods. Values are expressed as relative levels using the value normalized to the expression level of the internal control gene (Vero E6 β-actin). The relative quantification level using the value of PBS-treated control cells as a basal reference level for comparison is shown as the mean ± SD. * *p* < 0.05 and ** *p* < 0.01 indicate significant differences between groups

Both a high level of viral Ag-specific Ab as well as virus-neutralizing Ab in immune sera are critically correlated with effective virus protection after viral Ag-specific immune induction [18, 19]. Consequently, we measured the neutralizing activity of the Ab induced after immunization with the HBD 2-conjugated Ag (Fig. 5). First we assessed whether sera obtained from mice immunized with HBD 2-conjugated S RBD could inhibit the binding of the cognate Ag to its specific receptor-expressing cells. When we monitored the binding efficiency of S RBD on MERS-CoV receptor hDPP4-expressing Huh-7 cells, we found that the sera from mice immunized with HBD 2-conjugated S RBD efficiently inhibited the binding of the Ag compared to those obtained from mice immunized with either PBS or S RBD without HBD 2 conjugation (Fig. 5a). We next performed the receptor binding inhibition assay on the MERS-CoV-susceptible Vero E6 cell line using sera obtained from mice immunized with PBS, S RBD, or HBD 2-conjugated S RBD (Fig. 5b). As shown by the flow cytometry data, S RBD protein bound efficiently to the surfaces of Vero E6 cells (red line in upper left panel). Importantly, incubation with sera from HBD 2-conjugated S RBD mice showed almost complete inhibition of S RBD binding to cell surfaces (blue line in lower right panel) compared with that from mice immunized with S RBD alone (ca. 38% inhibition, sky-blue line in lower left panel). In contrast, sera from PBS-immunized mice did not exhibit significant inhibition of S RBD binding to the target cell surfaces (green line in upper right panel).

Finally, we examined whether the MERS-CoV load to the host cell was efficiently inhibited by immunized sera from mice. When we measured the transcript level of the upE gene of MERS-CoV in Vero E6 cells after infecting the virus that had been preincubated with mice sera via qRT-PCR, we found that the sera from mice immunized with HBD 2-conjugated S RBD displayed significantly higher inhibitory activity on MERS-CoV upE gene expression compared to those from mice immunized with either control PBS or S RBD without HBD 2 conjugation (*p* < 0.01 and *p* < 0.05, respectively; Fig. 5c). These results indicate that HBD 2-conjugated S RBD is superior to unconjugated S RBD in inducing the neutralizing Ab against MERS-CoV infection and is capable of promoting the protective immune response against virus infection. We conclude that HBD 2 could be used as an adjuvant to induce efficient innate and protective adaptive immunity against a viral Ag.

## Discussion

Human defensins are effector peptides produced by various cell types and have broad antibacterial, antiviral, and antifungal activity [20]. Defensins are small cysteine-rich cationic and amphipathic proteins. Their antiviral and antibacterial activity was originally attributed to their lipid perturbation activity because the disruption of viral glycoprotein function by membrane lipid perturbation leads to the inhibition of receptor binding and fusion of the enveloped virus to host cells. However, the observation that several classes of non-enveloped viruses are also sensitive to defensins led to the discovery of additional antiviral mechanisms of defensins [7]. Although additional mechanisms include receptor downregulation and the disruption of early events in viral infection, a universal mechanism for the neutralization of non-enveloped viruses remains elusive. In addition to their antibacterial and antiviral activity, defensins are one of the strongest central and peripheral defenders against pathogen infection, particularly in linking innate and adaptive immunity against pathogen infection through leukocytes, such as DCs and T cells [9]. In addition, a potent adjuvant is needed to enhance the immunogenicity and protective efficacy of subunit vaccines against pathogens [21, 22]. We thus investigated the adjuvant effects of defensins on antiviral innate and pathogenic Ag-specific adaptive immune responses using HBD 2 to analyze its potential use in the development of a subunit vaccine with self-adjuvant activity.

HBD 2-conjugated Ag treatment of macrophage-like THP-1 cells enhanced the transcript levels of antiviral molecules, including IFN-β, IFN-γ, MxA, PKR, RNaseL, and NOD2, and primary immune response-regulating cytokines and chemokines, although the induction level varied (Figs. 1, 2 and 3). IFNs are divided into type I and type II IFNs. Like IFN-γ, a type II IFN, type I IFNs, such as IFN-β, are induced by viral infection and confer antiviral activity to the host [23, 24]. IFNs exert their antiviral activity by enhancing the expression of numerous genes with antiviral activity, including MxA, a cytoplasmic GTPase that inhibits the replication of several RNA viruses [25, 26]. Double-stranded RNA-dependent protein kinase PKR and endogenous ribonuclease RNaseL are also antiviral effector proteins [25]. In contrast to MxA, the activation of PKR requires its binding to double-stranded RNA, and the activation of PKR leads to translational arrest via the phosphorylation of an essential translation initiation factor. This in turn activates the endogenous ribonuclease RNase L, which subsequently cleaves single-stranded RNAs, including mRNAs of viral and host cell origin [25].

We found that NOD2 expression was activated by HBD 2-conjugated Ag treatment of THP-1 cells (Fig. 3c). NOD2 was recently identified as a bacterial receptor that contributes to crosstalk between innate and adaptive immune systems in the digestive tract [27, 28]. NOD2 also functions as a cytoplasmic viral pattern recognition receptor by triggering the activation of IFN-regulatory factor 3 (IRF3) and the production of IFN-β [29]. NOD2 is expressed in immune cells, such as monocytes/macrophages, T cells, granulocytes, and DCs, as well as in colon epithelial cells [30, 31] and is required for the expression of a subgroup of intestinal antimicrobial peptides, such as cryptdins [28]. It is interesting that NOD2-deficient mice are susceptible to pathogen infection via the oral route but not intravenous or peritoneal routes. These reports indicate that NOD2 is essential for activating adaptive immunity by acting as an adjuvant receptor for Ab production, either directly or by enhancing the production of defensins [32, 33] or other immunostimulatory molecules. In addition, β-defensins have been reported to function as chemoattractants for various antigen-presenting cells (APCs), including immature DCs and macrophages, through chemokine receptors CCR6 and CCR2 [9, 34, 35]. Besides recruiting APCs to the inflammation site, β-defensins modulate the adaptive immune response by activating signal transduction through pattern recognition receptors for APC maturation, leading to the production of Th1-polarized cytokines and other important immunomodulatory factors. β-defensins also modulate the adaptive immune response by self-destructive signaling to eliminate activated APCs and prevent harmful effects of long-activated DCs and macrophages [36, 37]. Therefore, some β-defensins may counter suppressive pathogen-derived factors by generating robust host primary immune responses, thereby inducing an efficient protective immune response. Collectively, we presume that HBD 2 is able to stimulate not only antiviral innate immune responses, but also specific adaptive immune responses through enhanced delivery of fused antigens to APCs in vivo. Consequently, we assessed the ability of HBD 2 to promote Ag-specific immune response induction using the HBD 2-conjugated S RBD of MERS-CoV.

To confirm the adjuvant ability of HBD 2, we measured the inducing level of Ag-specific IgG after immunization with S RBD alone or with the HBD 2-conjugate and performed receptor-binding and viral inhibition assays to detect and quantify serum-neutralizing Abs to MERS-CoV using a viral receptor-expressing host cell-based assay. As expected, similar to the results of Ag-specific Ab within the respective immune sera, higher virus-neutralized activity was observed in sera obtained from HBD 2-conjugated S RBD-immunized mice compared to mice treated with S RBD alone or PBS only (Fig. 5). We also noted that the magnitude of the inhibitory effects of sera from HBD 2-conjugated Ag-immunized mice was generally higher, similar to the pattern of enhanced induction of antiviral innate immunity in HBD 2-conjugated Ag-treated THP-1 cells.

## Conclusions

In summary, we conclude that HBD 2 activates the primary antiviral innate immune response and may also mediate the induction of an effective adaptive immune response against a conjugated Ag. Our results indicate that HBD 2 can be used as an effective antiviral vaccine adjuvant and will provide useful material for further research on antiviral innate immune responses.

## Abbreviations

Ab: Antibody
Ag: Antigen
AMP: Antimicrobial peptide
APC: Antigen-presenting cell
DC: Dendritic cell
DMEM: Dulbecco’s Modified Eagle’s Medium
ELISA: Enzyme-linked immunosorbent assay
FBS: Fetal bovine serum
HBD: Human β-defensin
hDPP4: Human dipeptidyl peptidase-4
IFN: Interferon
MERS-CoV: Middle East respiratory syndrome-coronavirus
PBS: Phosphate-buffered saline
PCR: Polymerase chain reaction
PFU: Plaque-forming unit
qRT-PCR: Quantitative real-time polymerase chain reaction
RBD: Receptor-binding domain
S: Spike
SD: Standard deviation
upE: Upstream E

## References

[1] Lee S, Nguyen MT (2015). Recent advances of vaccine adjuvants for infectious diseases. Immune Netw.

[2] Aoshi T (2017). Modes of action for mucosal vaccine adjuvants. Viral Immunol.

[3] Ko EJ, Lee YT, Lee Y, Kim KH, Kang SM (2017). Distinct effects of monophosphoryl lipid a, oligodeoxynucleotide CpG, and combination adjuvants on modulating innate and adaptive immune responses to influenza vaccination. Immune Netw.

[4] Kuroda E, Coban C, Ishii KJ (2013). Particulate adjuvant and innate immunity: past achievements, present findings, and future prospects. Int Rev Immunol.

[5] Castañeda-Sánchez JI, Domínguez-Martínez DA, Olivar-Espinosa N, García-Pérez BE, Loroño-Pino MA, Luna-Herrera J, Salazar MI (2016). Expression of antimicrobial peptides in human monocytic cells and neutrophils in response to dengue virus type 2. Intervirology.

[6] Diamond G, Beckloff N, Weinberg A, Kisich KO (2009). The roles of antimicrobial peptides in innate host defense. Curr Pharm Des.

[7] Wilson SS, Wiens ME, Smith JG (2013). Antiviral mechanisms of human defensins. J Mol Biol.

[8] Oppenheim JJ, Yang D (2005). Alarmins: chemotactic activators of immune responses. Curr Opin Immunol.

[9] Yang D, Chertov O, Bykovskaia SN, Chen Q, Buffo MJ, Shogan J, Anderson M, Schröder JM, Wang JM, Howard OM, Oppenheim JJ (1999). Beta-defensins: linking innate and adaptive immunity through dendritic and T cell CCR6. Science.

[10] Chertov O, Michiel DF, Xu L, Wang JM, Tani K, Murphy WJ, Longo DL, Taub DD, Oppenheim JJ (1996). Identification of defensin-1, defensin-2, and CAP37/azurocidin as T-cell chemoattractant proteins released from interleukin-8-stimulated neutrophils. J Biol Chem.

[11] Ma C, Wang L, Tao X, Zhang N, Yang Y, Tseng CK, Li F, Zhou Y, Jiang S, Du L (2014). Searching for an ideal vaccine candidate among different MERS coronavirus receptor-binding fragments–the importance of immunofocusing in subunit vaccine design. Vaccine.

[12] Daigneault M, Preston JA, Marriott HM, Whyte MK, Dockrell DH (2010). The identification of markers of macrophage differentiation in PMA-stimulated THP-1 cells and monocyte-derived macrophages. PLoS One.

[13] Du L, Kou Z, Ma C, Tao X, Wang L, Zhao G, Chen Y, Yu F, Tseng CT, Zhou Y, Jiang S (2013). A truncated receptor-binding domain of MERS-CoV spike protein potently inhibits MERS-CoV infection and induces strong neutralizing antibody responses: implication for developing therapeutics and vaccines. PLoS One.

[14] Lu X, Whitaker B, Sakthivel SK, Kamili S, Rose LE, Lowe L, Mohareb E, Elassal EM, Al-sanouri T, Haddadin A, Erdman DD (2014). Real-time reverse transcription-PCR assay panel for Middle East respiratory syndrome coronavirus. J Clin Microbiol.

[15] Agrawal AS, Garron T, Tao X, Peng BH, Wakamiya M, Chan TS, Couch RB, Tseng CT (2015). Generation of a transgenic mouse model of Middle East respiratory syndrome coronavirus infection and disease. J Virol.

[16] Tseng CT, Huang C, Newman P, Wang N, Narayanan K, Watts DM, Makino S, Packard MM, Zaki SR, Chan TS, Peters CJ (2007). Severe acute respiratory syndrome coronavirus infection of mice transgenic for the human angiotensin-converting enzyme 2 virus receptor. J Virol.

[17] Yoshikawa N, Yoshikawa T, Hill Y, Huang C, Watts DM, Makino S, Milligan G, Chan T, Peters CJ, Tseng CT (2009). Differential virological and immunological outcome of severe acute respiratory syndrome coronavirus infection in susceptible and resistant transgenic mice expressing human angiotensin-converting enzyme 2. J Virol.

[18] Belshe RB, Newman FK, Cannon J, Duane C, Treanor J, Van Hoecke C, Howe BJ, Dubin G (2004). Serum antibody responses after intradermal vaccination against influenza. N Engl J Med.

[19] Quan FS, Yoo DG, Song JM, Clements JD, Compans RW, Kang SM (2009). Kinetics of immune responses to influenza virus-like particles and dose-dependence of protection with a single vaccination. J Virol.

[20] Lehrer RI, Lu W (2012). α-Defensins in human innate immunity. Immunol Rev.

[21] Nandre RM, Ruan X, Duan Q, Sack DA, Zhang W (2016). Antibodies derived from an enterotoxigenic *Escherichia coli* (ETEC) adhesin tip MEFA (multiepitope fusion antigen) against adherence of nine ETEC adhesins: CFA/I, CS1, CS2, CS3, CS4, CS5, CS6, CS21 and EtpA. Vaccine.

[22] Nandre R, Ruan X, Lu T, Duan Q, Sack D, Zhang W (2018). Enterotoxigenic *Escherichia coli* adhesin-toxoid multiepitope fusion antigen CFA/I/II/IV-3xSTa_N12S_-mnLT_G192G/L211A_-derived antibodies inhibit adherence of seven adhesins, neutralize enterotoxicity of LT and STa toxins, and protect piglets against diarrhea. Infect Immun.

[23] Kotenko SV, Gallagher G, Baurin VV, Lewis-Antes A, Shen M, Shah NK, Langer JA, Sheikh F, Dickensheets H, Donnelly RP (2003). 2003. IFN-lambdas mediate antiviral protection through a distinct class II cytokine receptor complex. Nat Immunol.

[24] Müller U, Steinhoff U, Reis LF, Hemmi S, Pavlovic J, Zinkernagel RM, Aguet M (1994). Functional role of type I and type II interferons in antiviral defense. Science.

[25] Goodbourn S, Didcock L, Randall RE (2000). Interferons: cell signalling, immune modulation, antiviral response and virus countermeasures. J Gen Virol.

[26] Haller O, Frese M, Kochs G (1998). Mx proteins: mediators of innate resistance to RNA viruses. Rev Sci Technol.

[27] Gutierrez O, Pipaon C, Inohara N, Fontalba A, Ogura Y, Prosper F, Nunez G, Fernandez-Luna JL (2002). Induction of Nod2 in myelomonocytic and intestinal epithelial cells via nuclear factor-kappa B activation. J Biol Chem.

[28] Kobayashi KS, Chamaillard M, Ogura Y, Henegariu O, Inohara N, Nuñez G, Flavell RA (2005). Nod2-dependent regulation of innate and adaptive immunity in the intestinal tract. Science.

[29] Sabbah A, Chang TH, Harnack R, Frohlich V, Tominaga K, Dube PH, Xiang Y, Bose S (2009). Activation of innate immune antiviral responses by Nod2. Nat Immunol.

[30] Ogura Y, Inohara N, Benito A, Chen FF, Yamaoka S, Nunez G (2001). Nod2, a Nod1/Apaf-1 family member that is restricted to monocytes and activates NF-kappaB. J Biol Chem.

[31] Ogura Y, Lala S, Xin W, Smith E, Dowds TA, Chen FF, Zimmermann E, Tretiakova M, Cho JH, Hart J, Greenson JK, Keshav S, Nuñez G (2003). Expression of NOD2 in Paneth cells: a possible link to Crohn’s ileitis. Gut.

[32] Tani K, Murphy WJ, Chertov O, Salcedo R, Koh CY, Utsunomiya I, Funakoshi S, Asai O, Herrmann SH, Wang JM, Kwak LW, Oppenheim JJ (2000). Defensins act as potent adjuvants that promote cellular and humoral immune responses in mice to a lymphoma idiotype and carrier antigens. Int Immunol.

[33] Oppenheim JJ, Biragyn A, Kwak LW, Yang D (2003). Roles of antimicrobial peptides such as defensins in innate and adaptive immunity. Ann Rheum Dis.

[34] Biragyn A, Surenhu M, Yang D, Ruffini PA, Haines BA, Klyushnenkova E, Oppenheim JJ, Kwak LW (2001). Mediators of innate immunity that target immature, but not mature, dendritic cells induce antitumor immunity when genetically fused with nonimmunogenic tumor antigens. J Immunol.

[35] Röhrl J, Yang D, Oppenheim JJ, Hehlgans T (2010). Human beta-defensin 2 and 3 and their mouse orthologs induce chemotaxis through interaction with CCR2. J Immunol.

[36] Biragyn A, Ruffini PA, Leifer CA, Klyushnenkova E, Shakhov A, Chertov O, Shirakawa AK, Farber JM, Segal DM, Oppenheim JJ, Kwak LW (2002). Toll-like receptor 4-dependent activation of dendritic cells by β-defensin 2. Science.

[37] Biragyn A, Coscia M, Nagashima K, Sanford M, Young HA, Olkhanud P (2008). Murine beta-defensin 2 promotes TLR-4/MyD88-mediated and NF-κB-dependent atypical death of APCs via activation of TNFR2. J Leukoc Biol.

